# Selective recognition of human telomeric G-quadruplex with designed peptide *via* hydrogen bonding followed by base stacking interactions†

**DOI:** 10.1039/c9ra08761c

**Published:** 2019-12-04

**Authors:** Shikhar Tyagi, Sarika Saxena, Nikita Kundu, Taniya Sharma, Amlan Chakraborty, Sarvpreet Kaur, Daisuke Miyoshi, Jadala Shankaraswamy

**Affiliations:** Structural Biology Lab, Amity Institute of Biotechnology, Amity University Uttar Pradesh Sector-125, Expressway Highway Noida 201303 India ssaxena1@amity.edu sarikaigib@yahoo.co.in +91-120-4735600; Department of Chemical Engineering, Monash University Clayton VIC 3800 Australia; Faculty of Frontiers of Innovative Research in Science and Technology (FIRST), Konan University 7-1-20 Minatojima-minamimachi, Chuo-ku Kobe Hyogo 650-0047 Japan; Department of Fruit Science, College of Horticulture, Mojerla, Sri Konda Laxman Telangana State Horticultural University 509382 Telangana India

## Abstract

We described a novel synthetic peptide in which a glutamine residue binds through hydrogen bonding to a guanine-base and a trytophan residue intercalates with K^+^ resulting in stabilization of a human telomeric G-quadruplex with high selectivity over its complementary c-rich strand and a double-stranded DNA and its complementary C-rich strand. This peptide offers great potential for cancer treatment by inhibiting the telomere extension by telomerase.

## Abstract 2

A new synthetic peptide is presented. A Glu residue binds through H-bonding to a guanine-base and a Trp residue intercalates with K^+^ resulting in stabilization of a human telomeric G-quadruplex with high selectivity over a complementary c-rich strand and double-stranded DNA.

## Graphical abstract

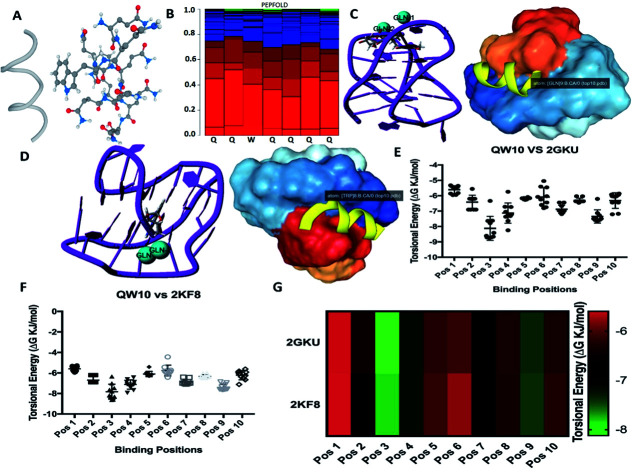

## Introduction

G-quadruplexes (G4s) are non-canonical stable secondary structures found in G rich nucleic acids wherein guanine bases associate to form tetrastranded structures *via* Hoogsteen hydrogen bonds that stack in a planar arrangement, a G-quartet, stabilized in the central position of the cavity due to the binding of K^+^ or Na^+^ ions.^1,2^ There is evidence which shows the over representation of G4 forming sequences in the upstream promoter region of various oncogenes and at the 3′-telomeric ends of eukaryotic chromosomes. In recent years, the emergence of strong evidence related to the existence, function and biological role of G4 in cellular environments has contributed to enormous interest.^3–6^ Telomeres are shortened with each successive replication due to the end replication problem and play an important role in chromosomal integrity. Cancer cells have higher expression of telomerase and are closely associated with the cellular immortality of more than 80% of human cancer cells. Small ligands which bind and stabilize the telomere structure have been recognized to be promising targets for anticancer drugs.^7–9^ In past few years; efforts are directed towards the development of G4 binding ligands with increasing specificity and selectivity for different strand orientation and loop length.^10–12^ Much research has focussed on reporting ligands having a planar aromatic surface which is accessible for G4 binding by π-stacking interactions.^11^ To further increase the selectivity and the affinity of a ligand, efforts are towards the incorporation of neutral or cationic side chain which binds in the grooves or loops of the G4 structure by means of electrostatic as well as hydrogen-bonding interactions.^10^ However, higher selectivity of the ligand to bind with G4 in the presence of excess amounts of double-stranded DNA is still a major challenge.^12^ Not only by small size molecules, but also middle size molecules such as a peptide could be useful to increase affinity and specificity in G4 bindings.

**Scheme 1:**
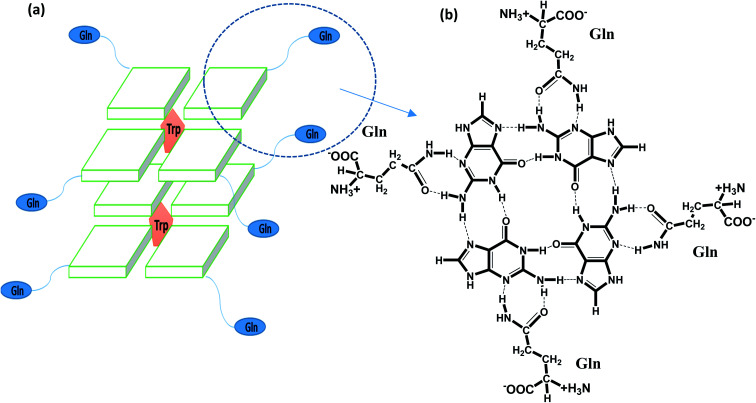
Schematic representation of recognition of glutamine with G-base in G-quadruplex by hydrogen bonding followed by intercalation of tryptophan within G-quartet plane (a) and hydrogen bonding of glutamine with G base (b).

We address these issues, by using a designed peptide, QW10(QQWQQQQWQQ), which may be possible to bind with telomeric DNA G4 with high selectivity. In the peptide, we incorporate glutamine (Q) residues to present both hydrogen bonding donor and acceptor sites with guanine bases in a G-quartet plane (as shown in Scheme 1a and b) as well as the backbone phosphates and ribose rings. These hydrogen bonding donors and acceptors also provide water solubility of the peptide. Moreover, tryptophan (W) residues were incorporated to provide an aromatic rings for π–π stacking interactions that can fit within the G-quartet planes, as demonstrated in recent studies.^11–16^ In contrast to previous small molecules and peptide targeting DNA,^17^ we did not introduce positive residues such as arginine and lysine, to reduce a non-specific binding with other DNA structures, including DNA duplex. Based on the molecular design, it is possible to consider that binding modes of QW10 are hydrogen bonding and stacking interaction. The present binding studies reveals important hints on the relationship between the structure and the selective binding of the peptide as promising class of new G4 ligands.

## Materials and methods

### Materials

DNA oligonucleotide of PAGE purified grade, were purchased from Helix Biosciences (Delhi, India) and controlled peptide [QQWQQQQWQQ] was synthesized by standard F-moc Chemistry on the solid phase. The peptide was purified by HPLC and the purity was confirmed by MALDI-TOF-MS. The concentration of the peptide was determined by measuring the absorbance of Trp at the C-terminal at 280 nm at 25 °C. Single-strand concentrations of DNA oligonucleotides were determined by measuring the absorbance at 260 nm at a high temperature using a Shimadzu 1800 Spectrophotometer (Schimadzu, Tokyo, Japan) connected to a thermoprogrammer. Single-strand extinction coefficients were calculated from mononucleotide and dinucleotide data using the nearest-neighbour approximation.^18–20^

### Circular dichroism spectroscopy

CD spectra were carried out on JASCO-715 spectropolarimeter using a quartz cuvette of 1 cm path length. All the spectra were recorded in the range of 200–350 nm wavelengths at a scanning rate of 100 nm min^−1^. Before measurement, the samples were heated to 95 °C in water bath and slowly cooled till water attains room temperature and incubated at 4 °C overnight to avoid any non-equilibrium structures. Average scans of the DNA samples were subtracted from the buffer scan and data was normalized as a function of DNA strand concentration and pathlength of the cuvette. The CD curve was plotted between ellipticity as a function of wavelength. The molar ellipticity change at 295 nm *vs.* the DNA concentration was fitted to the following equation for one binding site to evaluate the value of dissociation constant.*y* = *a* × [peptide]^*n*^/(*K*_d_^*n*^ + [peptide]^*n*^) + 1

### Thermal melting analysis

UV absorbance of different samples were recorded with a Shimadzu 1800 spectrophotometer (Shimadzu, Tokyo, Japan) equipped with a temperature controller. Melting curves of DNA structures were obtained by measuring the UV absorbance at 260 or 295 nm in buffer pH 7.0 [100 mM NaCl or 100 mM KCl, and 0.5 mM EDTA] in the presence or absence of QW10 peptide at DNA : peptide ratio (1 : 0), (1 : 1), (1 : 2), (1 : 5), and (1 : 10). The *T*_m_ values for 4 μM DNA structures were obtained from the UV melting curves as described previously.^19^ The heating rates were 0.5 °C min^−1^. The thermodynamic parameters were evaluated from the fit of the melting curves to a theoretical equation for an intramolecular association as described previously.^19,20^ Before measurement, the samples were heated to 95 °C in water bath and slowly cooled till water attains room temperature and incubated at 4 °C overnight to avoid any non-equilibrium structures. Experiment has been repeated in triplicates to reproduce the data.

### Native gel electrophoresis

For doing native gel experiment, 15% (w/v) polyacrylamide gel was used. Here in PAGE experiment, samples were composed of 30 mM sodium cacodylate buffer (pH 7.4), 100 mM KCl and 0.5 mM EDTA. The samples were heated to 95 °C in water bath and slowly cooled till water attains room temperature and incubated at 4 °C overnight. The running buffer TBE (pH 7.4) also contains the same concentration of salt and EDTA as in gel and oligonucleotide sample. Experiment was performed in cold room at constant 50 V. A 1 : 1 mixture of glycerol and orange-G was used for tracking the movement of DNA oligonucleotides in the gel. Finally, gel was stained using silver staining and imaged using Gel-Doc (Biorad, Gurgaon, Haryana, India).

### Fluorescence measurements

Fluorescence experiments were performed by utilizing a JASCO FP 8300 spectrofluorometer (JASCO, Tokyo, Japan). Experiments were carried out at 25 °C in a 3 mm path-length quartz cuvette for 4 μM peptide in pH 7.0 buffer containing 100 mM KCl, 0.5 mM EDTA titrated with equimolar concentration of HTPu. The temperature of the cell holder was regulated by a JASCO ETC-273T temperature controller. Samples were prepared by same procedure. Excitation and emission slit width were 5 nm each and the samples were excited at 275 nm and the emission was recorded in a range of 300 nm to 500 nm. Experiment has been repeated in triplicates to reproduce the data. Modified Stern–Volmer equation was used to analyze fluorescence quenching data to find out various binding parameters for this interaction since binding parameters are vital to study about the binding mechanism.
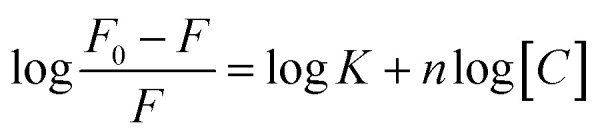
where the highest fluorescence intensity in the absence of ligand is *F*_0_ whilst *F* depicts fluorescence intensity in the presence of ligand, *K* depicts the binding constant, *n* depicts the number of binding sites, and the concentration of RT is depicted by *C*.

## Results

### Designing of peptide

QW10 (QQWQQQQWQQ) is a designed peptide with an abundance of glutamine with intermittent tryptophan residues. Basic idea of designing this peptide was to make them structure selective based on the hydrogen bonding binding ability of side chain of glutamine with the available hydrogen bonding sites of the guanine base after the G-quadruplex formation. The carbonyl group and amino group in the side chain of glutamine may recognize the G-base of G-quadruplex in sequence and structure specific manner followed by the intercalation of tryptophan residues.

### Molecular docking studies of QW10 with crystal structures of human telomeric G-quadruplexes

**Fig. 1:**
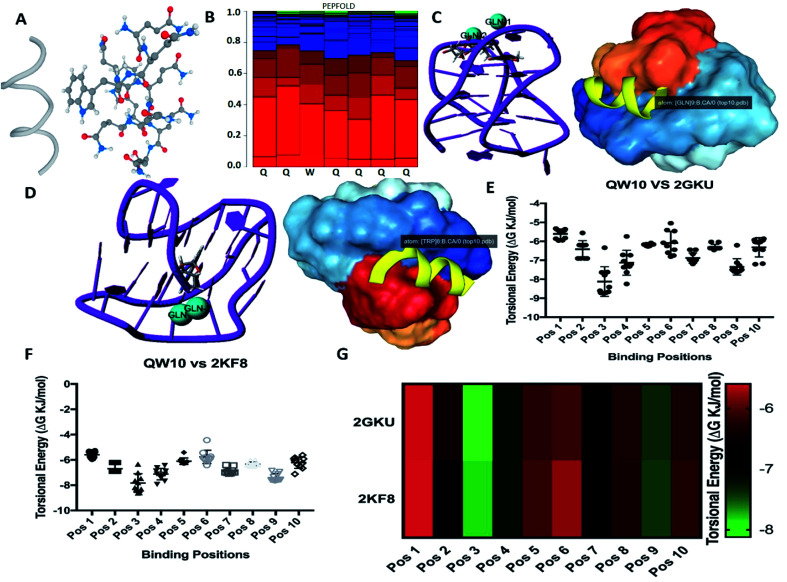
Interaction of QW10 *versus* 2GKU and 2KF8. (A) Helical structure and molecular model of the peptide QW10, (B). SA peptide profile of QW10. The probabilities at each position of the sequence, of the 27 SA letters are the sorted from helical (red), coil (blue) to extended (green). (C) QW10 *versus* 2GKU cartoon model showing the interacting GLN residues and the surface structure of 2GKU showing the binding pocket holding QW10, (D) QW10 *versus* 2KF8 cartoon model showing the interacting GLN residues and the surface structure of 2KF8 showing the binding pocket holding QW10, (E). Torsional energy (Δ*G*, KJ mol^−1^) *versus* different binding positions in 2GKU, (F). Torsional energy (Δ*G*, KJ mol^−1^) *versus* different binding positions in 2KF8, (G). Torsional energy profile of 2GKU and 2KF8 showing different binding positions interacting with QW10. Red showing less binding energy while green showing high binding energy, therefore more stability.

**Fig. 2:**
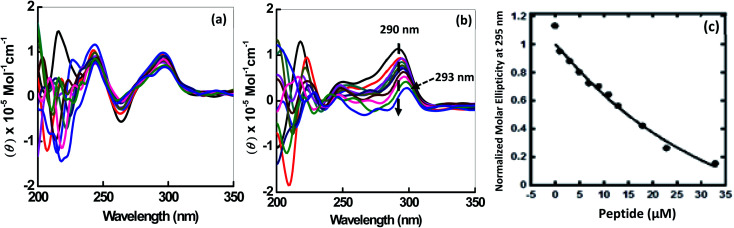
CDspectra of 2 μM HTPu in buffer containing NaCl (100 mM) (a) and 100 mM KCl (b), 0.5 mM EDTA, without any additive (black line) and titrated with an increase in concentration of QW10 peptide. Normalized molar ellipticity of 2 μM HTPu with increasing concentration of QW10 peptide (c).

We selected a human telomeric G4 as a target; because of detailed structures have been reported.^21,22^ To investigate the most probable binding mode of QW10 with G4, we performed molecular docking studies with two crystal structures of human telomeric G-quadruplex (2GKU)^21^ and (2KF8).^22^ The structure of QW10 was predicted using PEP-Fold structural alphabet (SA) prediction profiling which describes the conformations of four consecutive residues. PEP-Fold works on the principle of prediction of each fragment of four residues in a query to perform a 3D assembly of the complete structure using a greedy algorithm and the sOPEP coarse-grained force field. On the other hand, a mixed (3 + 1) strand fold topology, G4 (2GKU) and a basket type intramolecular G4 (2KF8) used as target G4s. Torsional binding energy for QW10 was investigated for 2KF8 and 2GKU using HPEPDOCK and MTi AutoDock to confirm the highest binding energies. The locations where QW10 can interact with the G4 were mapped with the top ten torsions in terms of their binding energy (Δ*G* (−)ve). The individual structural maps for the ten torsions with rotatable bonds were evaluated for both quadruplexes. The SA profile of QW10 demonstrates 80% helical structure of the peptide (Fig. 1A and B). The next model possible is a coil model but has less possibility in the biological context. Hence, a helical QW10 was used for our docking studies (Fig. 1A). Interactions between QW10 *versus* 2GKU (Fig. 1C) and 2KF8 (Fig. 1D) was quantified and ranked for the top ten positions of their binding energy (Δ*G* (−)ve) (Fig. 1E and F). However, for specific interactions position 3 was used to study the interactions (Fig. 1C and D). The different locations of the peptide interacting with the G-quadruplex showed higher degree of interactions at position 3 which was mapped to be GLN8,9 (for both 2GKU and 2KF8) followed by TRP8 (Fig. 1C and D). In both cases the torsions for this structure had the highest number of rotational bonds (6/molecule of QW10) giving more stability in the environment for interacting with the QW10-G4 complexes (Fig. 1G). In both the quadruplexes, the repeating GLN residues in the middle of the peptide resulted in an induced fit for interacting with the quadruplex. Resulting in that both the G4 were similar in interaction with QW10 (Fig. 1G). Based on the docking results, we proposed the schematic representation of the binding mode of the QW10 with G-quadruplex unit in which side chain of the glutamine binds with guanine by hydrogen bonding and tryptophan intercalates between them (Scheme 1).

### Effect of monovalent ions (Na^+^ or K^+^) on the human telomeric G4 with and without peptide

CD spectroscopy was employed to investigate the changes on the conformation of human telomeric G-quadruplexes (HTPu 5′-GGGTTAGGGTTAGGGTTAGGGTTA-3′) upon peptide binding. The structure of each DNA strand is in 30 mM sodium cacodylate buffer pH (7.0) and 100 mM Na^+^ or 100 mM K^+^ in presence and absence of peptide (Fig. 2). CD spectrum in 100 mM Na^+^ is characterized by a positive peak at 290 nm and negative peak at 260 nm, typically observed for an antiparallel G-quadruplex in the presence of Na^+^.^23,24^ Next, HTPu was titrated with increasing concentrations of QW10 in the presence of 100 mM Na^+^ (Fig. 2a). We observed a slight decrement of CD intensity at 290 nm and small shoulder around 270 nm upon the titration of QW10. However, these changes are very small and the overall CD spectra are almost similar. These results indicate that QW10 binds to the antiparallel G4 is not significantly altered by the binding of QW10.^23^ In contrast, HTPu in the presence of K^+^ (Fig. 2b), exhibits a strong positive peak around 290 nm with a shoulder around 255 nm, and a smaller negative peak at 240 nm, indicating a formation mixed G-quadruplex, consistent with the previously published report.^24,25^ On titrating HTPu with peptide, we observed a decrement of CD signal. In addition, we observed that the positive peak at 290 nm shifts to 293 nm and that the 254 nm peak merges towards 280 nm. These changes indicate that the binding of peptide inducing structural change. We proposed that the gradual decrease in CD intensity on increasing the peptide concentration is due to the aggregation of the QW10-G4 complex. This possibility will be further explored by native gel electrophoresis data in the following section.

Understanding binding affinity which is strength of the binding interaction between the DNA and peptide is a key to understand the intermolecular interactions, as a part of the drug discovery to check their binding efficiency with their targets selectively and specifically. Fig. 2c shows plot of CD intensity at 290 nm *vs.* QW10 concentration in the presence of K^+^. The CD intensity change at 290 nm was fitted to a theoretical equation with an assumption of a one to one binding to evaluate half concentration (EC_50_). The values of EC_50_ were evaluated to be 39.5 ± 0.2 μM in the presence of K^+^ respectively at 25 °C. Note that these EC_50_ are not dissociation constants of the complex, because there is no isodichroic point in the CD spectra during the titration experiments, indicating that there are multiple states in the system. The EC_50_ values indicates that the high concentration of the QW10 are required to occupy HTPu G4 binding sites. This may be due to the two way binding of the peptide with G-quadruplex structure, one is hydrogen bonding involving the side chain of the glutamine with G-bases and another is due to the intercalation of the tryptophan residues within G-quartet core. The possible role of glutamine is consistent with poly-Q diseases in which an elongation of continuous glutamine residues leads to protein aggregations. Interestingly, it is reported that glutamine accelerates liquid–liquid phase separation of proteins and protein–nucleic acid complexes.^26^ Moreover, tryptophan residues are most important for TAR DNA binding protein 43 to undergo liquid–liquid phase separation.^27^ Therefore, the results obtained here showing a large complex formation of QW10-HTPu is consistent with the previous studies indicating importance of glutamine and tryptophan.

### Thermodynamic analysis of the human telomeric G-quadruplex structure with and without peptide

**Fig. 3:**
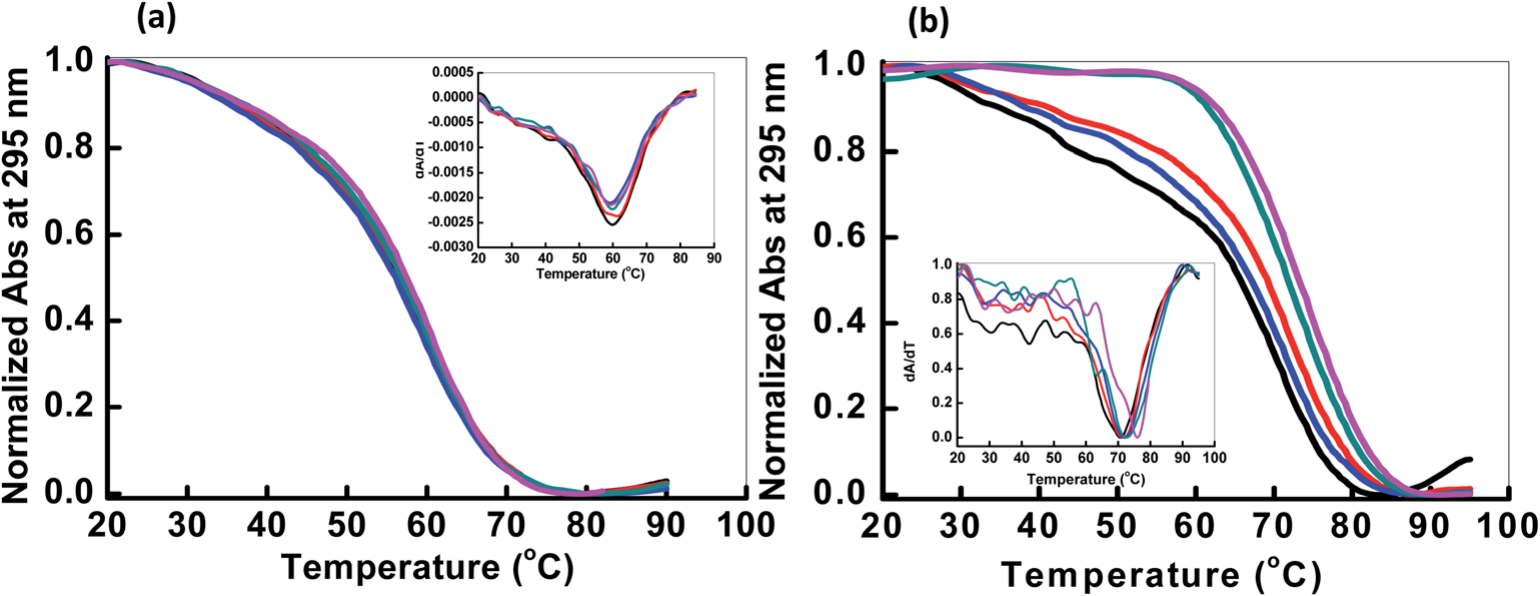
Normalized UV melting curves of 4 μM HTPu in buffer containing NaCl (100 mM) (a) and 100 mM KCl (b), 0.5 mM EDTA, without any additive (black line); HTPu : QW10 ratio (1 : 1) (red line), (1 : 2) (blue line), (1 : 5) (green line), (1 : 10) (pink line). Experiment has been repeated in triplicates to reproduce the data.

Next, we explored the thermal stability of the DNA structures with and without peptide. Fig. 3 shows normalized UV melting profile of 4 μM HTPu in the buffer containing 100 mM NaCl or KCl in the absence and presence of QW10. The ratios of HTPu : QW10 are (1 : 0, 1 : 1, 1 : 2, 1 : 5 and 1 : 10) respectively (Fig. 3). The melting temperature (*T*_m_) was evaluated by a curve fitting procedure as described previously.^19,20^ The *T*_m_ of the HTPu G4 was slightly increased from 61.5 °C, 61.5 °C, 62.0 °C, 62.5 °C and 63.0 °C in the presence of 100 mM Na^+^ with the DNA peptide ratio of (1 : 0, 1 : 1, 1 : 2, 1 : 5 and 1 : 10) respectively. The melting curves with a single transition and overall 1 °C difference in the *T*_m_ values in different DNA : peptide ratios. These results are consistent with that HTPu maintains the antiparallel G4 on increasing the peptide concentration in the presence of Na^+^ as shown above.

On the contrary of the *T*_m_ values in the presence of Na^+^, the *T*_m_ of the HTPu G4 was more significantly varied from (68.0 °C, 68.5 °C, 70 °C, 71.5 °C and 73.5 °C) in the presence of 100 mM K^+^ in DNA peptide ratio of (1 : 0, 1 : 1, 1 : 2, 1 : 5 and 1 : 10) respectively. These results indicated that these G4s possess similar thermal stability in the presence of Na^+^. On the other hand, the *T*_m_ value of the HTPu G4 was increased from 68 °C to 73.5 °C in the presence of K^+^, therefore, the G4 is significantly stabilized by QW10. This indicates the initial recognition of the peptide at low concentration to the mixed G-quadruplex and its preferential binding to antiparallel G4 higher concentration as shown and discussed above in CD and in Native PAGE results in the following section. We also prepared the Watson–Crick base paired duplex (5′-GGGTTAGGGTTAGGGTTAGGGTTA-3′ purine strand and 3′-TAACCCTAACCCTAACCCTAACCC-5′ pyrimidine strand) by mixing purine and pyrimidine rich strand in 1 : 1 ratio and checked the thermal stability with (1 : 10) and without peptide (Fig. S1†). The *T*_m_ value of the duplex was 66.5 °C and decreased to 61.5 °C after the addition of DNA : peptide in 1 : 10 ratio indicating the destabilization of the duplex on peptide binding. We further the thermal stability of pyrimidine rich strand (HTPy) with (1 : 10) and without peptide. We observed a marginal change in *T*_m_ value of the HTPy with and without peptide. HTPy has *T*_m_ value 52.6 °C which decreased to 51.1 °C upon peptide binding (Fig. S2†). These results of the DNAs forming other structures suggest that the binding of QW10 is in a structure specific manner, although further systematic studies are required.

To assess the origin of the observed stabilities of HTPu G4 upon the complex formation with QW10, the thermodynamic parameters of their formations, such as the enthalpy change (Δ*H*°), the entropy change (Δ*S*°), and the free energy change at 25 °C 
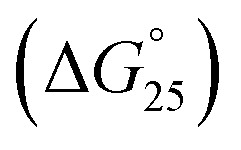
 of the HTPu G4 formation were estimated in the presence and absence of 4 to 40 μM peptide (summarized in Table 1). On increasing peptide concentration from 4 μM to 40 μM in maintaining the DNA: peptide ratios of (0 : 1, 1 : 1, 1 : 2, 1 : 5 and 1 : 10) in a buffer containing 100 mM NaCl and 30 mM sodium cacodylate buffer (pH 7.0), Δ*H*° decreased −17.2 kcal mol^−1^, −17.6 kcal mol^−1^, −18.3 kcal mol^−1^, −19.3 kcal mol^−1^, −22.5 kcal mol^−1^, *T*Δ*S*° decreased from −51.3 kcal mol^−1^, −52.5 kcal mol^−1^, −54.5 kcal mol^−1^, −57.5 kcal mol^−1^, −66.5 kcal mol^−1^. The free energy (Δ*G*°) at 298 K follows the same order. 
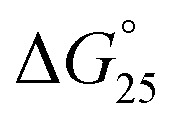
 decreased −0.5 kcal mol^−1^, −0.7 kcal mol^−1^, −0.7 kcal mol^−1^, −1.0 kcal mol^−1^, −1.7 kcal mol^−1^, however, in a K^+^ containing buffer, Δ*H*° decreased −20.9 kcal mol^−1^, −26.2 kcal mol^−1^, −26.4 kcal mol^−1^, −26.7 kcal mol^−1^, −27.3 kcal mol^−1^, *T*Δ*S*° decreased from −61.3 kcal mol^−1^, −75.7 kcal mol^−1^, −76.1 kcal mol^−1^, −78.2 kcal mol^−1^, −79.6 kcal mol^−1^. 
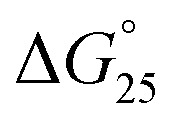
 decreased −0.8 kcal mol^−1^, −0.8 kcal mol^−1^, −1.1 kcal mol^−1^, −1.5 kcal mol^−1^, −2.0 kcal mol^−1^. Therefore, stabilization of the HTPu G-quadruplex by the binding of peptide is promoted by a favourable an enthalpic contribution exceeding an unfavorable entropy change. Accordingly, specific intermolecular hydrogen bonding between the glutamine residues and G4, as well as the stacking interactions of tryptophan may contribute this enthalpic stabilization of G4. These enthalpic stabilization effects on G4 derived from specific interactions have been reported for small molecular ligands effects G4s.^28,29^

Thermodynamic parameters for HTPu-QW10 interaction studies in the presence of 100 mM Na^+^ and K^+^^a^

**Table:** 

Na^+^				
Abbreviations	*T*_m_/°C	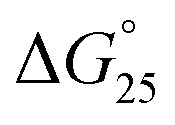 /kcal mol^−1^	Δ*H*°/kcal mol^−1^	*T*Δ*S*°/kcal mol^−1^
HTPu : QW10 (1 : 0)	61.5	−0.5 ± 0.2	−17.2 ± 0.6	−51.3 ± 1.2
HTPu : QW10 (1 : 1)	61.5	−0.7 ± 0.1	−17.6 ± 0.2	−52.5 ± 2.0
HTPu : QW10 (1 : 2)	62.0	−0.7 ± 0.2	−18.3 ± 0.3	−54.5 ± 1.6
HTPu : QW10 (1 : 5)	62.5	−1.0 ± 0.3	−19.3 ± 2.2	−57.5 ± 1.8
HTPu : QW10 (1 : 10)	63.0	−1.7 ± 0.2	−22.5 ± 1.2	−66.5 ± 1.4

a All experiments were carried out in a buffer containing 100 mM NaCl, 100 mM KCl, 30 mM sodium cacodylate buffer (pH 7.0), and 0.5 mM EDTA. Thermodynamic parameters are the average values obtained from melting curves replicated three times.

**Table:** 

K^+^				
Abbreviations	*T*_m_/°C	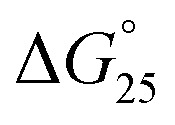 /kcal mol^−1^	Δ*H*°/kcal mol^−1^	*T*Δ*S*°/kcal mol^−1^
HTPu : QW10 (1 : 0)	68.0	−0.8 ± 0.3	−20.9 ± 0.4	−61.3 ± 1.2
HTPu : QW10 (1 : 1)	68.5	−0.8 ± 0.1	−26.2 ± 0.2	−75.7 ± 2.4
HTPu : QW10 (1 : 2)	70.0	−1.1 ± 0.2	−26.4 ± 1.2	−76.1 ± 1.6
HTPu : QW10 (1 : 5)	71.5	−1.5 ± 0.3	−26.7 ± 1.6	−78.2 ± 1.8
HTPu : QW10 (1 : 10)	73.5	−2.0 ± 0.2	−27.3 ± 2.2	−79.6 ± 3.1

### Higher order structure of the telomere DNA with the peptide

**Fig. 4:**
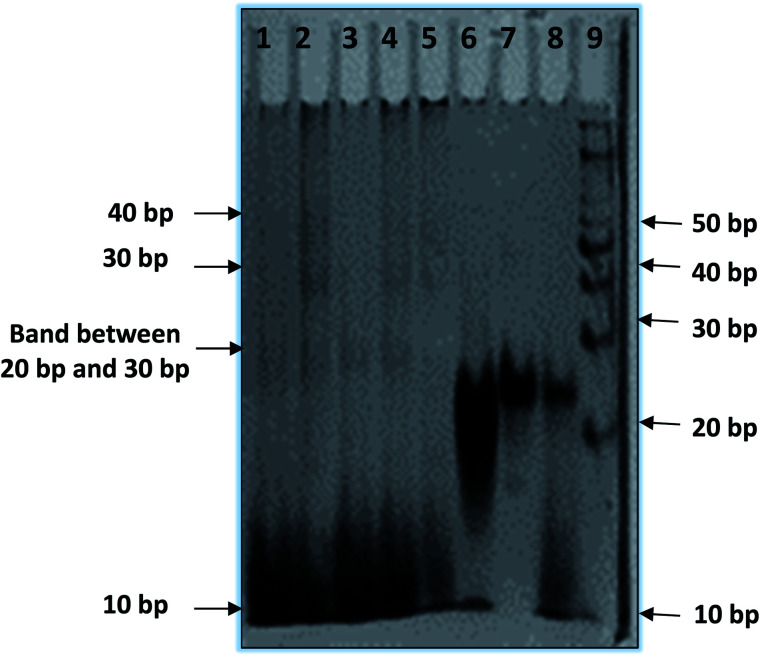
Native gel electrophoresis of 15 μM HTPu in (a) 30 mM sodium cacodylate buffer (pH 7), 100 mM KCl, 1 mM EDTA. HTPu : QW10 Lane 1 (1 : 0), Lane 2 (1 : 1), Lane 3 (1 : 10), Lane 4 (1 : 30), Lane 5 (1 : 100) respectively. Lane 6 (PAL20) intramolecular palindromic sequence used as marker (PAL20), Lane 7 HTPu·HTPy (duplex) : QW10 (1 : 0), Lane 8 HTPu·HTPy (duplex) : QW10 (1 : 100), Lane 9 – 10 bp ladder.

In order to reveal a molecular mechanism of the HTPu and QW10 binding and possible aggregation of the complex, we further investigated the complex in the presence of K^+^ using non-denaturating PAGE (Fig. 4). The PAGE experiment can discriminate molecularity of HTPu and HTPu-QW10 complex. The electrophoretogram in Fig. 4 shows the structural status of HTPu in the presence and absence of QW10. 10 bp DNA ladder and control size markers like PAL20 were used to compare their electrophoretic mobility. PAL 20 is a palindromic sequence which moved as a 40-mer duplex in non-denaturating PAGE. The Lane 1 of Fig. 4 displayed one band which migrated equivalent to 10 base pairs, corresponding 20 nucleotides band indicating that HTPu folds into unimolecular structure. Next, we checked the migration of HTPu in the presence of QW10. The HTPu : QW10 are in the ratio of (1 : 1) (Lane 3), (1 : 20) (Lane 4), (1 : 30) (Lane 5), and (1 : 100) (Lane 6) respectively. We observed three bands between 20 to 30 bp, 30 bp and 40 bp respectively on comparing with 10 bp band in all four lanes (Lane 4 to Lane 6). This observation leads to the possibility that peptide binds with HTPu, stabilize the structure and appeared in the form of higher order multimeric DNA-peptide complexes which is consistent with our CD data. Now, focussing on the upper bands between 20 to 30 bp, 30 bp and 40 bp, we proposed that as glutamine in its side chain contains carbonyl and amino group, so it might be possible that few free glutamine units may be acting as linker to attach G4 units together by end to end association bonding. In lanes 5 and 6, it can be clearly seen that lower band intensity has decreased and increased in upper bands. Interestingly, it has been perceived that HTPu got stuck up at the top of the well in both the lanes 5 and 6, which is visible as a darker region at the edge of the well. This gives the possibility of forming a higher order structure. On mixing purine and pyrimidine (HTPu·HTPy) in 1 : 1 ratio, we observed a single band which migrated close to 20 bp indicating the formation of duplex. In the presence of peptide, two bands appeared corresponding to 10 bp and 20 bp. This indicates that the duplex is destabilized in the presence of peptide in which lower band at 10 bp corresponds to dissociated single strand bound with peptide while the upper band at 20 bp is undissociated duplex. These results suggest that QW10 peptide has differential effect on hydrogen bonded DNA duplex and Hoogsteen bonded G-quadruplex.

### Fluorescence measurement of human telomeric DNA with and without peptide

The binding affinity of peptide to HTPu G-quadruplex was also investigated by fluorescence measurements. Upon excitation at 275 nm, the peptide produced an emission band due to the presence of tryptophan residue with a maxima centered at 347 nm (Fig. S4†). The intramolecular HTPu G quadruplex in the presence of potassium cations was added to the peptide until very small changes in fluorescence spectra were observed. The fluorescence of QW10 was quenched without a peak shift on increasing the DNA concentration indicating that tryptophan is intercalating within G-quadruplex planes during binding. Intrinsic fluorescence is often employed to characterize ligand binding and also to find various parameters associated with this binding. Quenching in fluorescence is universally observed phenomenon wherein a decrease in the fluorescence takes place in the presence of ligand *viz*. with increasing concentration of ligand there is an observed quenching of the fluorescence and this quenching can be retorted to find various binding parameters. When a complex is formed between G-quadruplex and the peptide, binding constant for this complex obtained through quenching studies is implicative of the strength of this interaction. Fluorescence quenching analysis revealed that binding constant is of the order 10^6^ M^−1^. The binding constant obtained from modified Stern–Volmer plot is 2.4 × 10^6^ M^−1^ and binding constant value of such high order is implicative of strong binding between G-quadruplex and the peptide.

## Conclusions

In conclusion, the biological significance of G-quadruplexes has been well recognized and has been emerged as attractive candidates for cancer therapy. Human telomeric G quadruplex forming sequence folds into multiple G-quadruplex conformations. Thus, the discovery and development of small molecules that can interact with telomere G-quadruplex DNA and stabilize the G quadruplex structures may provide necessary opportunities for telomerase inhibition. In this manuscript the conformational polymorphism of the DNA human telomeric repeat sequence and its interaction with peptide has been investigated by CD and verified by computational approach. Our results allowed us to discriminate the binding of peptide with hydrogen bonded DNA duplex and Hoogesteen bonded G-quadruplex. We observed significant changes in CD spectra on titrating the Human telomere quadruplex with QW 10 peptide. Significant changes in molar ellipticity clearly indicate that peptide is binding to the G-quadruplex. Changes in molar ellipticity were observed in presence of both monovalent ions used during studies, but significant changes were observed in the presence of potassium in comparison to sodium which indicates that the binding of peptide is conformation specific. As the structure of telomere G-quadruplex is different in K^+^ and Na^+^, therefore, peptide recognizes and binds to these structures differently. We observed a significant stabilization on the binding of peptide in the presence of K^+^ in comparison to Na^+^. Overall, there was 5.5 °C in the *T*_m_ values at DNA : peptide (1 : 0 and 1 : 10) respectively. This indicates that peptide is binding to the G-quadruplex and stabilizing the structure which is further confirmed by the presence of higher molecular weight G-quadruplex peptide complexes observed in Native PAGE Data. Fluorescence data also supports the binding of peptide with DNA as discussed above. Based on CD, UV-thermal melting, Native PAGE and fluorescence studies we conclude that the peptide can be used as a drug molecule for the recognition of G-quadruplex to inhibit telomerase activity and thereby, offers a new approach for cancer therapeutic intervention.

## Conflicts of interest

There are no conflicts to declare.

## Supplementary Material

RA-009-C9RA08761C-s001
