# Supplementary material for: Selective recognition of human telomeric G-quadruplex with designed peptide via hydrogen bonding followed by base stacking interactions
Source: RSC Adv. 2019 Dec 4;9(69):40255–62. doi: 10.1039/c9ra08761c (PMC9076235; doi:10.1039/c9ra08761c)
Supplement: RA-009-C9RA08761C-s001 [file RA-009-C9RA08761C-s001.pdf]

Electronic Supplementary Material (ESI) for RSC Advances.
This journal is © The Royal Society of Chemistry 2019

Selective Recognition of Human Telomeric G-quadruplex with Designed Peptide via Hydrogen Bonding Followed by Base Stacking Interactions

Shikhar Tyagi[a], Sarika Saxena[a*], Nikita Kundu[a], Taniya Sharma[a], Amlan Chakraborty[b], Sarvpreet Kaur[a], Daisuke Miyoshi[c], Jadala. Shankaraswamy[d]

[1]Amity Institute of Biotechnology, Amity University Uttar Pradesh, Noida 201313, India.
[2]Department of Chemical Engineering, Monash University, Clayton, VIC 3800, Australia.
[3]Faculty of Frontiers of Innovative Research in Science and Technology (FIRST), Konan University, 7-1-20 Minatojima-minamimachi, Chuo-ku, Kobe, Hyogo 650-0047, Japan
[4]Department of Fruit Science, College of Horticulture, Mojerla, Sri Konda Laxman Telangana State Horticultural University, 509382, Telangana, India.
Note: Shikhar Tyagi[1], Nikita Kundu[1] and Taniya Sharma[1] share equal contribution.

Address of Correspondence: Structural Biology Lab, Sector-125, Expressway Highway, Amity Institute of Biotechnology, Amity University Uttar Pradesh, Noida 201313, India. Phone Number: 0120-4735600, Email: ssaxena1@amity.edu;sarikaigib@yahoo.co.in.

Page 1: Contents

Page 2: Figure S1 for the Normalized UV melting curve of HTPu.HTPy duplex with and without peptide in K^+.

Page 2: Figure S2 for the Normalized UV melting curve of HTPy with and without peptide in K^+.

Page 3: Figure S3 for the emission spectra of QW10 with increasing concentration of HTPu.

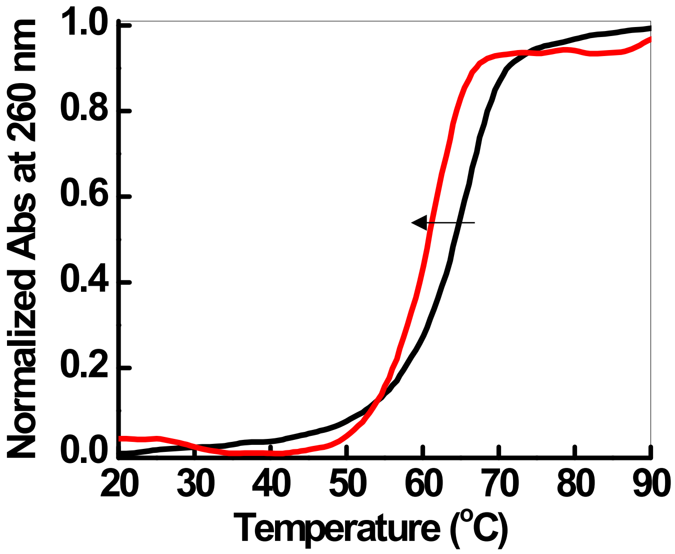

Figure S1 NormalizedUV melting curves of 4μMHTPu.HTPyin buffer containing 100 mMKCl, 0.5 mM EDTA, without any additive (black line); HTPu.HTPy: QW10 ratio (1:10) (red line).

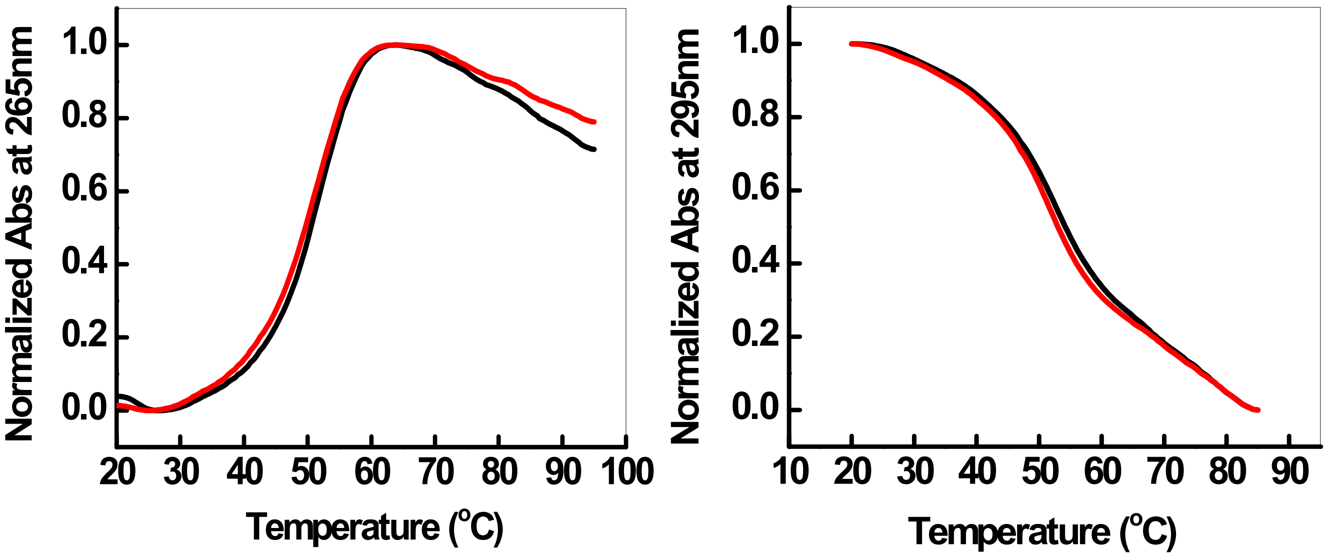

Figure S2 NormalizedUV melting curves of 4μMHTPyin buffer containing 100 mMKCl, 0.5 mM EDTA, without any additive (black line); HTPu.HTPy: QW10 ratio (1:10) (red line) at 265 nm (a) and at 295 nm (b).

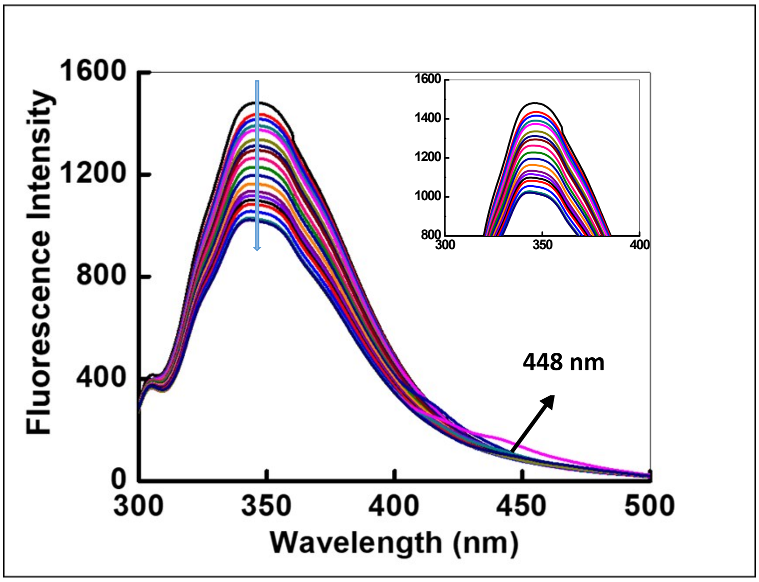

Figure S3a Emission Spectra of QW10 peptide with HTPu in buffer (pH 7.0) containing 100 mM KCl, 0.5 mM EDTA, at 25°C. QW10=4 µM titrated with equimolar HTPu stock in an increasing concentration.

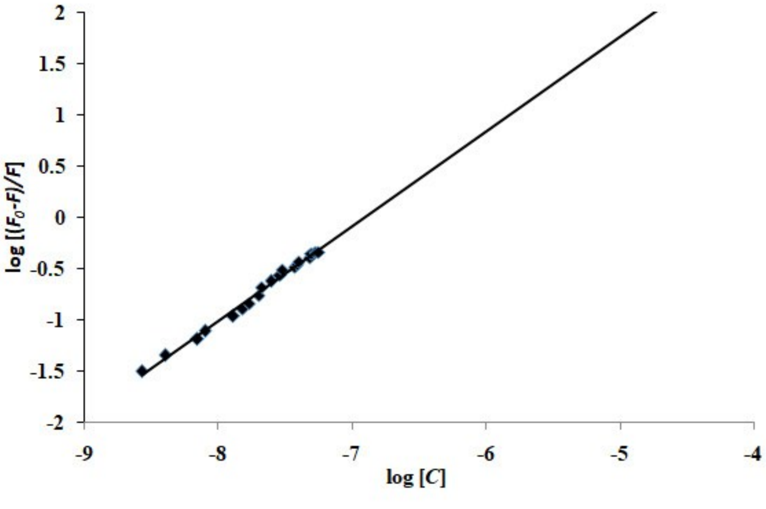

Figure S3b: Modified Stern Volmer plot. The experimental data fitting of double log relation to find binding parameters.
